# Endoscopic vacuum therapy (eVAC) combined with continuous perianastomotic irrigation for prevention of anastomotic leak after surgical ampullectomy

**DOI:** 10.1007/s00423-024-03408-7

**Published:** 2024-07-18

**Authors:** Olga Meier (Adamenko), Carlo Ferrari, Jonas Peter Ehrsam, Annamaria Porreca, Stefan Seewald, Stefan Groth, Jean-Pierre Gutzwiller, Jan Schmidt

**Affiliations:** 1 Hirslanden Hospitals, Kappelistrasse, 7, Zürich, 8002 Switzerland; 2 Università degli Studi di Milano, Milan, Italy; 3 Department of Medical, Oral and Biotechnological Sciences, University “G. D’Annunzio”, via dei Vestini, Chieti, 66100 Italy; 4 GastroZentrum Hirslanden, Zürich, Switzerland; 5 Magendarm Thalwil AG, Thalwil, Switzerland; 6 University of Nicosia, Medical School, Nicosia, Cyprus

**Keywords:** Ampullectomy, Leak, Prophylactic, Preemptive, Vacuum, Fistula

## Abstract

**Purpose:**

Transduodenal surgical ampullectomy (tAMP) with papillary reimplantation is a valid alternative to pancreaticoduodenectomy for lesions of the periampullary region not amenable to endoscopic resection. As tAMP is burdened by high rates of biliopancreatic-enteric anastomotic leak, we tested preventive endoluminal vacuum therapy (eVAC) combined with post-operative continuous perianastomotic irrigation (CPI) to reduce such anastomotic leak.

**Methods:**

Between 10/2013 and 09/2023, 37 patients undergoing laparotomic tAMP (with or without jejunal transposition) and papillary reimplantation at Hirslanden Klinik Zurich were retrospectively analysed; of these, 16 received prophylactic eVAC combined with CPI, while the remaining represented the historical cohort.

**Results:**

The eVAC-CPI-group and the historical-cohort were homogeneous in demographic characteristics. Surgery in the prophylactic eVAC-CPI-group lasted about 30 min longer due to eVAC application (*p* = 0.008). The biliopancreatico-enteric anastomotic leak rates were 6.2% in the eVAC-CIP-group vs. 19.0% in the historical-cohort (*p* = 0.266). Along, a strong trend of less severe post-operative complications in general (*p* = 0.073), and borderline-significantly less cases of acute pancreatitis (*p* = 0.057) and tAMP-related re-operations or re-interventions (*p* = 0.057) in particular, were observed in the eVAC-CPI-group. The only anastomotic leak in the eVAC-CPI-group was successfully managed through repeated cycles of eVAC. The device was well tolerated by all patients; no vacuum/irrigation-related complications or malfunctioning occurred.

**Conclusion:**

Our study is the first to provide some technical insights demonstrating the safety and feasibility of a prophylactic approach with eVAC and perianastomotic irrigation to reduce anastomotic leak after tAMP. Increasing the number of subjects will confirm the benefit of our promising results.

## Introduction

Tumours of the periampullary region account for 5% of all gastrointestinal tract malignancies [1]. They belong to four different entities, whether they originate from pancreatic duct, mucosa of the ampulla of Vater, common distal bile duct or duodenum [1]. Carcinogenesis in adenomas of the papilla is believed to follow the same adenoma-to-carcinoma pathway as in the development of colon cancer [2]. The frequency of malignant foci within an ampullary adenoma on surgical specimens may be as high as 47%, causing endoscopic biopsies to be inaccurate to rule out intra-adenomatous malignancies due to their high false-negative rates [3, 4]. 

Pancreaticoduodenectomy (PD), transduodenal surgical ampullectomy (tAMP), and endoscopic ampullectomy (eAMP) are currently available options [3]. The growing body of literature regarding tAMP and eAMP reflects the interest toward less invasive approaches compared to PD for the treatment of benign and early malignen tumors of the ampulla of Vater [5]. Surgical tAMP is generally preferred in case of benign ampullary lesions not amenable to endoscopic treatment (e.g. larger than 4 cm or ingrowth for > 1 cm into bile/pancreatic duct), without malignant features (e.g. soft, non-friable and non-ulcerated lesions), abdominal exploration for another indication, or in elderly patients with comorbid conditions precluding more invasive resections [5]. 

The overall, pooled surgical complication rate after tAMP is 28.3%, with individual studies ranging between 7.7 and 68% [6]. The three major forms of postoperative morbidity are represented by acute pancreatitis (10 − 50% of cases), haemorrhage (3.8 − 25% of cases), sometimes necessitating emergency reoperation and wound infection (5 − 20.7% of cases) [6]. The same analysis [6] reported an overall mortality of 0.9% after tAMP. However, in our clinical experience, a strong determinant of postoperative morbidity and prolonged length of stay after tAMP is represented by dehiscence of the delicate bilio-pancreatico-enteric anastomosis, leading to postoperative pancreatic fistula (POPF) or bilio-pancreatic fistula and its associated potentially life-threatening conditions. This event can be relatively frequent in this type of surgery because tAMP usually deals with small main pancreatic ducts and soft pancreatic parenchyma, which are two among the best acknowledged risk factors for POPF after pancreatic surgery.

Hence, we present some technical insights and initial results on safety and feasibility of prophylactic endoluminal vacuum therapy (eVAC) combined with post-operative continuous perianastomotic irrigation (CPI) applied in patients undergoing tAMP for peripampullary lesions, in the attempt to reduce the bilio-pancreatic leak (AL) after papillary reimplantation. We also compare surgical outcomes with historical controls who underwent tAMP before introducing prophylactic eVAC as standard intraoperative measure.

## Materials and methods

### Patient selection

This retrospective, observational study was conducted at the Department of General Surgery, Hirslanden Klinik and Klinik Im Park (Zurich, Switzerland). All patients undergoing tAMP between October 2013 and September 2023 were included; all surgeries, intra- and post-operative endoscopies as well as postoperative care were performed by investigators, experienced in hepatobilopancreatic surgery or endoscopy. The postoperative course was managed according to a standard care protocol. Postoperative complications were graded according to the Clavien-Dindo classification [7]. Data retrieval and study protocol were approved by the local institutional review board (BASEC-Nr. 2018 − 00183).

### Statistical analysis

Descriptive statistics were expressed as median and interquartile range (IQR) for continuous variables, and as absolute frequency (column percentage) for categorical ones. Association between categorical variables was investigated using Pearson’s Chi-squared test (for cell frequency *n* ≥ 5) and Fisher’s exact test (for cell frequency *n* < 5). Normality of distribution was tested using the Shapiro-Wilk’s test. When parametric assumptions were met, Student’s two-tailed t-test was used to compare the means of continuous variables; otherwise, the Mann–Whitney test was performed. All statistical tests were 2-sided, with a significance level set at *p* < 0.05. Statistical analysis was performed with SPSS^®^ version 29 (IBM, Chicago, IL, USA).

### Surgical procedure - resection phase

**Fig. 1:**
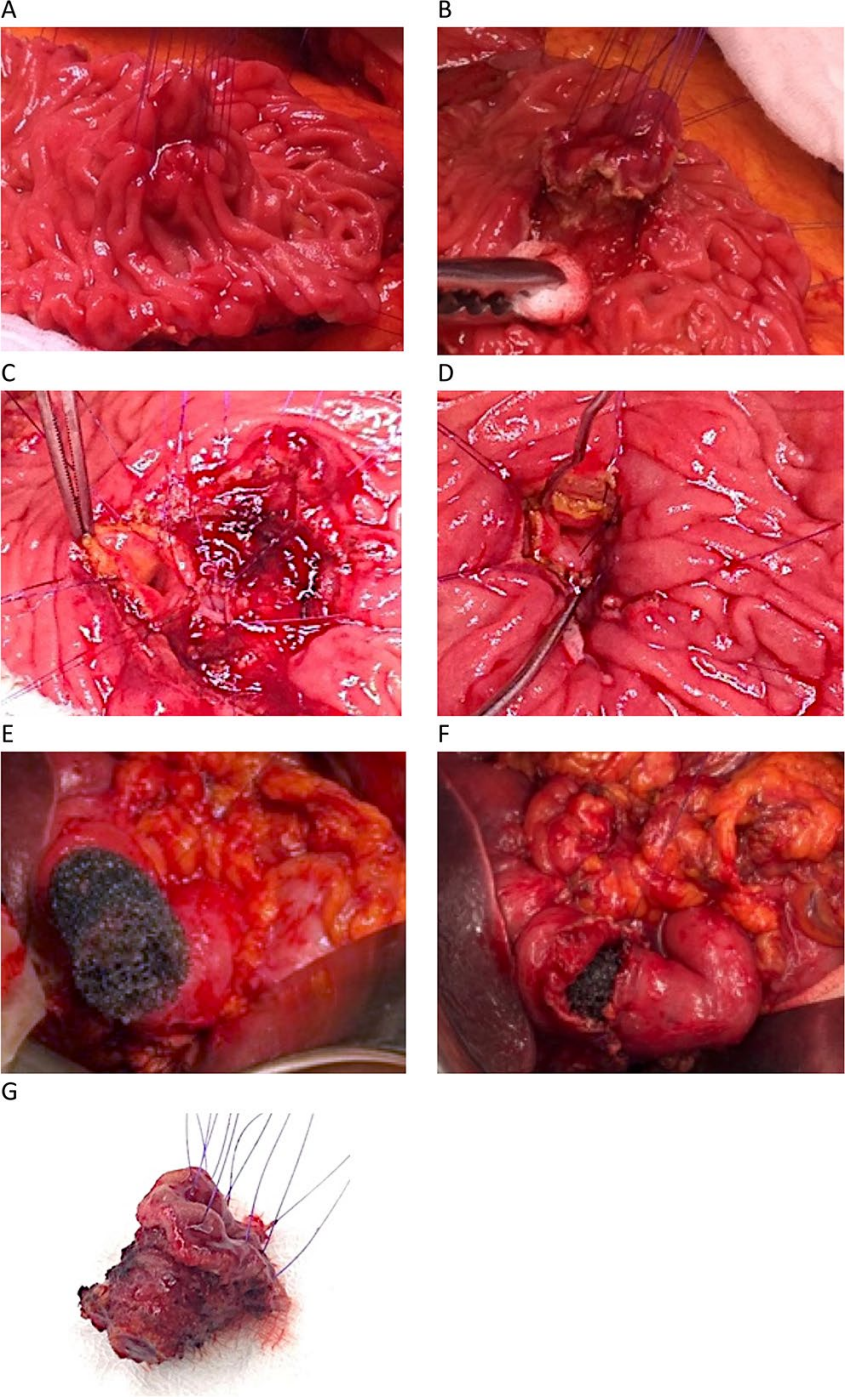
Example of a typical surgical ampullectomy and reconstruction along with placement of prophylactic endoluminal vacuum therapy (eVAC): (**A**) Encircled papillary area in the incised duodenum. (**B**) Circular incision leaving a macroscopic free margin around the lesion. (**C**) Reconstruction by creation of a common bilio-pancreatic ostium. (**D)** Reimplantation of the common bilio-pancreatic ostium within the duodenal wall. (**E)** Transnasal endoscopic guided eVAC sponge positioning under direct view of the surgeon in front of the papilla reimplantation site. (**F)** Closure of the duodenotomy over the sponge. (**G**) Removed surgical specimen

A median laparotomy and a wide Kocher manoeuvre expose the second duodenal portion. A longitudinal incision along its lateral duodenal wall exposes the lesion. After retrograde cholecystectomy the main bile duct is probed through the cystic duct to expose the papillary area (Fig. 1A). A circular incision with an electrified needle leaves a 5 mm macroscopic free margin around the lesion (Fig. 1B). The surgical specimen is removed including the full-thickness duodenal wall (Fig. 1G). Once removed, a common bilio-pancreatic ostium is created with monofilament sutures (Fig. 1C).

Intraoperative frozen section is performed. For benign or in situ malignant lesions no lymphadenectomy is performed. In case of early invasive lesions, lymphadenectomy of pre- and retro-pancreatic, hepatoduodenal ligament, hepatic artery and celiac trunk lymphnodes is carried out. For locally-advanced lesions, the procedure is converted into a standard PD.

### Surgical procedure - reconstruction phase

In case of limited loss of substance within the peripapillary area, the common bilio-pancreatic ostium is reimplanted within the duodenal wall with 6 − 0 polydioxanone (PDS), duct-to-mucosa interrupted sutures (Fig. 1D). Patency of pancreatic and biliary duct is confirmed with a probe and by means of an intraoperative cholangiogram. No routine stents are left neither in the pancreatic nor in the biliary duct. The lateral duodenal wall is eventually closed with a 5 − 0, continuous, double layer, monofilament suture.

**Fig. 2:**
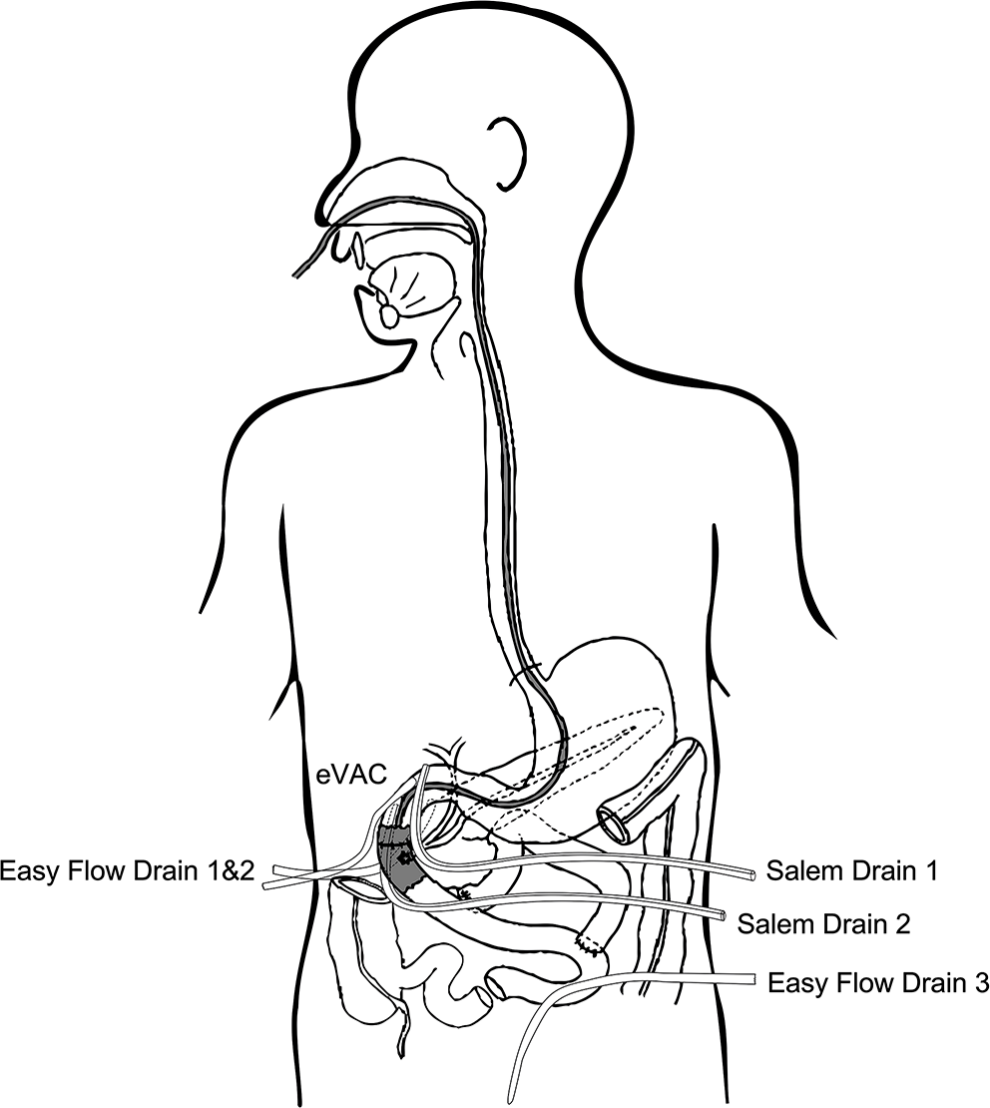
Schematic representation of reconstruction technique, prophylactic endoluminal vacuum therapy (eVAC) positioning, and perianastomotic irrigation (Salem)-drainage placement (CIP), after ampullectomy with jejunal transposition and papillary reimplantation

When a major duodenal wall resection makes a direct reconstruction impossible, the entire pars II with or without pars III of duodenum is resected en-block with the ampullary region. The second jejunal loop is transected 10 cm downstream the Treitz ligament: its efferent portion is transposed in the superior abdomen, in a retrocolic fashion, with the anti-mesenterial surface facing the common bilio-pancreatic ostium. A single-layer, 6 − 0 PDS, duct-to-mucosa anastomosis is performed between the common bilio-pancreatic ostium and the transposed jejunum. A termino-terminal, double-layer, 4 − 0 PDS, continuous duodeno-jejunal anastomosis restores the intestinal continuity. Eventually, the duodenal blind loop is anastomosed 40 cm downstream the papillary reimplantation site according to a Roux-en-Y reconstruction (Fig. 2).

### eVAC positioning

As illustrated in Fig. 2, an eVAC is inserted via the nasal route under endoscopic guidance. The eVAC sponge is positioned under direct view of the surgeon in front of the papilla reimplantation site (Fig. 1E and F).

### Irrigation-drainage positioning for CIP

Two irrigation-drainage Salem sump tubes are placed in front and behind the duodenotomy (or duodeno-jejunal anastomosis) and continuously flushed for CIP with 100 ml/h of Ringer solution for each drainage (Fig. 2). We have previously established this method [8] for the post-operative irrigation and drainage of the perianastomotic area in other major pancreatic resections, where it significantly contributed to reduction and improved healing of POPF.

### Post-operative management

The continuous suction pressure of eVAC is set at -75 mmHg. The negative pressure therapy is interrupted three times a day and the sponge line is flushed with 10 ml of saline solution in order to confirm patency of the system. Parenteral feeding is started on the first postoperative day (POD) and the patient can immediately drink up to 300 ml of water (which are, indeed, drained by eVAC). Between POD 5 and 7, depending on the availability of the endoscopist, the eVAC is removed under endoscopic guidance and the papillary anastomotic area is inspected to recognize signs of anastomotic leak. If negative, eVAC is removed, and the patient is allowed to gradually start feeding. In case of evidence of partial anastomotic dehiscence, a new eVAC is replaced and changed weekly until healing is complete. Removal of the CIP-drainages occurs depending on concentrations of pancreatic enzymes in the drained fluids as previously described [8].

## Results

Thirty-seven patients underwent tAMP during the study period. Of these, 16 received prophylactic eVAC of the anastomotic region combined with post-operative CPI; the remaining 21 represented the historical cohort. Detailed demographic, clinical and perioperative data of patients are presented in Table 1.

**Table 1:** Demographic, clinical and perioperative patients’ characteristics

Variables	Overall (*N* = 37)	Historical Cohort (*N* = 21)	eVAC-CIP Group (*N* = 16)	*p*
Age, years, median (q1; q3)	72.0 (56.5; 78.0)	71.0 (55.0; 76.5)	75.0 (60.5; 78.8)	0.404
BMI, kg/m^2^, median (q1; q3)	26.4 (24.0; 30.9)	25.8 (23.4; 31.2)	27.0 (24.3; 30.9)	0.728
Sex female, n (%)	17 (45.9)	11 (52.4)	6 (37.5)	0.508
ASA score, median (q1; q3)	2 (2;3)	2 (2;2)	2 (2;3)	0.165
1, n (%)	1 (2.7)	1 (4.8)	0	1.000
2	25 (64.9)	16(76.2)	9 (56.3)	0.291
3	11 (29.7)	4 (19.0)	7 (43.8)	0.151
Operative time, minutes, median (q1; q3)	158 (132; 187)	142 (116; 174)	175 (147; 229)	0.008
Lymphadenectomy, n (%)	11 (29.7)	5 (23.8)	6 (37.5)	0.475
Duodenal resection, n (%)	18 (48.6)	10 (47.6)	8 (50.0)	1.000
Blood losses, mL, median (q1; q3)	50 (50; 100)	50 (50; 100)	100 (50; 100)	0.130
eVAC^c^ removal, days, median (q1; q3)	-	-	6.0 (6.0; 6.0)	-
Continuous perianastomotic irrigation days, median (q1; q3)	-	-	11.5 (7.0; 15.0)	-
Postoperative complications, median (q1; q3)	II (0-IIIa)	II (0-IIIB)	0 (0-II)	0.073
0, n (%)	16 (43.2)	7 (33.3)	9 (56.3)	0.196
I	1 (2.7)	1 (4.8)	0	1.000
II	12 (32.4)	7 (33.3)	5 (31.3)	1.000
IIIA	1 (2.7)	0	1 (6.3)	0.432
IIIB	4 (10.8)	3 (14.3)	1 (6.3)	0.618
IVA	2 (5.4)	2 (9.5)	0	0.495
IVB	2 (5.4)	2 (9.5)	0	0.495
Types of postoperative complications				
Biliopancreatic-enteric anastomotic leak, n (%)	5 (13.5)	4 (19.0)	1 (6.3)	0.266
Acute pancreatitis	5(13.5)	5 (23.8)	0	0.057
Sepsis	3 (8.1)	3 (14.3)	0	0.243
Re-ICU	5(13.5)	5 (23.8)	0	0.057
Arrhythmia	3 (8.1)	1 (4.8)	2 (12.5)	0.568
Local minor hemorrhage	4 (10.8)	2 (9.5)^b^	2 (12.5)	1.000
Chylus leak	1 (2.7)	1 (4.8)	0	1.000
Paralytic ileus	4 (10.8)	2 (9.5)	2 (12.5)	1.000
Urinary retention	2 (5.4)	1 (4.8)^b^	1 (6.3)	1.000
Re-operation				
Biliopancreatic-enteric anastomotic leak^a^	2 (5.4)	2 (9.5)	0	0.495
Peripancreatic abscess^a^	1 (2.7)	1 (4.8)^b^	0	1.000
Aberrant bile duct leak	1 (2.7)	1 (4.8)	0	1.000
Abdominal wound dehiscence	2 (5.4)	2 (9.5)	0	0.495
Re-intervention				
Biliopancreatic-enteric anastomotic leak re-drain^a^	1 (2.7)	1 (4.8)	0	1.000
Local major hemorrhage embolization^a^	1 (2.7)	1 (4.8)	0	1.000
^a^Re-operation or re-Intervention related to tAMP	5(13.5)	5 (23.8)	0	0.057
Length of hospital stay, days, median (q1; q3)	12.0 (9.5; 18.0)	11.0 (9.0; 17.5)	13.0 (10.3; 18.8)	0.404
30-days hospital readmission, n (%)^b^	5 (13.5)	5 (23.8)	0	0.057
related to tAMP	2 (5.4)	2 (9.5)	0	0.495
Mortality, n (%)	0	0	0	1.000

^a^ Re-operation or re-Intervention related to tAMP

^b^ One of these occurences represents a single case of complication for 30-days hospital readmission

*Abbreviations* tAMP, transduodenal surgical ampullectomy; BMI, Body Mass Index; ASA, American Society of Anesthesiology; eVAC, endoluminal Vacuum Therapy; ICU, intensive care unit

The two groups were homogeneous in terms of age, Body Mass Index (BMI) and sex. Although not statistically significant, the prophylactic eVAC-CPI-group had slightly higher ASA scores with respect to the historical cohort. Rates of duodenal resection and lymphadenectomy were comparable between the groups. The median operative time was significantly longer in the prophylactic eVAC-CPI-group than in the historical cohort (175 min. vs. 142 min., *p* = 0.008), thus reflecting an average of 30 min for eVAC application.

Overall, we experienced 5 bilio-pancreatic leaks: 4 (19.0%) occurred in the historical group, while the remaining 1 (6.2%) happened after the introduction of the prophylactic eVAC-CPI method. Two leaks in the historical group required surgical revision with redo of the anastomosis on POD 1 and 14, respectively; the other two cases of the historical group were managed conservatively with prolonged drainage in place in one case and radiological drainage of intraabdominal fluid collection in the other case. The only anastomotic leak occurring in the prophylactic eVAC-CPI-group was successfully managed with prolonged drainage and repeated eVAC cycles, eventually leading to complete healing of the anastomosis. Among all 16 patients in the eVAC-CPI-group, we did not experience any vacuum-related complications, such as decubitus, malfunctioning or dislocation. One patient accidentally self-removed the eVAC on POD 2, during his stay in the ICU; however, the recover was uneventful, and he could be discharged home on POD 9.

Even though, the trend of a lower incidence of bilio-pancreatic leaks in the prophylactic eVAC-CPI-group was non-significantly different to the historical cohort per se (*p* = 0.266), the eVAC-CPI-group showed a strong trend of less severe post-operative complications measured by CD index (*p* = 0.073). More importantly, among these complications, acute pancreatitis (*p* = 0.073) and tAMP related re-operations or re-interventions were borderline-significantly reduced in the eVAC-CPI-group (*p* = 0.057 and *p* = 0.057, respectively). The incidence of readmission to ICU and the incidence of 30-day hospital readmission were also borderline-significantly lower in the eVAC-CPI-group (*p* = 0.057 and *p* = 0.091, respectively).

Morality rates were zero in both groups.

Histopathological findings of preoperative biopsies, intraoperative frozen sections and definitive postoperative histology of each patient are summarized in Table 2. In more than one third of cases (13/37) there was disagreement between the preoperative histology and the final, definitive pathological examination of the surgical specimen (Table 2). In particular, benign preoperative histology demonstrated foci of invasive disease (e.g. in situ carcinomas) after definitive pathological exam. In only three cases the preoperative biopsy overestimated the real malignancy of the neoplasia, with high-grade dysplasia not being confirmed at the definitive, postoperative histology.

**Table 2:** Histopathological characteristics

Patient No.	Preoperative biopsy	Frozen section	Final pathology
1	Adenoma with HGD	Adenoma with LGD	Adenoma with LGD
2	Adenoma with LGD	Adenoma with LGD	Adenoma with LGD
3	Adenoma with HGD	Adenoma	Adenoma with HGD
4	Adenoma with LGD	Adenoma with LGD	Adenoma with LGD
5	Adenoma with HGD	Adenoma with HGD	Adenocarcinoma [pT1a, V0, L0, R0]
6	Adenoma	No malignancy	Inflammation and fibrosis
7	Adenoma with HGD	Adenoma	Adenoma with LGD
8	GIST	GIST	GIST [pT3, pN0(0/2), V0, L0, R0]
9	Adenoma with LGD	Adenoma with LGD	Adenoma with LGD
10	Adenoma	Adenoma	Adenocarcinoma [pT1a, G1, V0, L0, Pn0, R0]
11	Adenoma	Adenoma with foci of adenocarcinoma	Adenocarcinoma [pT1b(sm3), pN0(0/11), G2, V0, L0, Pn1, R0]
12	Sarcoma recurrence with duodenal infiltration**	Sarcoma	Retroperitoneal sarcoma [rpT2, G3, R1]
13	GIST	GIST	GIST [pT2, pN0(0/5), V0, L0, Pn0, R0]
14	Adenoma with LGD	Adenoma with HGD	Adenoma with foci of HGD
15	Adenoma with LGD	Adenoma with LGD	Adenoma with LGD
16	GIST	GIST	GIST [pT2, V0, L0, Pn0, R0]
17	Adenoma with LGD	Adenoma with LGD	Adenoma with foci of HGD
18	Paracholedochal cyst (benign)	Fibrosis	Adenoma with LGD
19	Adenoma with LGD	Adenoma with HGD	Adenoma with foci of HGD
20	Adenoma with LGD	Adenoma with LGD	Adenoma with LGD
21	Adenoma with LGD	Adenoma with HGD	Adenoma with foci of HGD
22*	Adenocarcinoma	Adenocarcinoma [pT1b (sm2)]	Adenocarcinoma [pT1b (sm3), pN0, G2, V0, L0, Pn0, R0 (vm-, hm-)]
23*	Adenocarcinoma	Adenocarcinoma	Adenocarcinoma [pT3, pN0 (0/13), G3, L0, V0, Pn0, R0]
24*	Adenoma	Adenoma	Adenoma with foci of HGD
25*	Adenoma with HGD	Adenoma with HGD	Adenoma with HGD
26*	Adenoma with HGD	Adenoma with HGD	Adenoma with HGD
27*	Pyloric gland adenoma	No malignancy	Inflammatory pseudotumor (benign)
28*	Adenoma with LGD	Adenoma with LGD	Adenoma with LGD
29*	Adenoma with LGD	Adenoma with LGD	Adenoma with LGD
30*	Adenoma with LGD	Adenoma with LGD	Adenoma with LGD
31*	Adenoma with HGD	Adenoma	Adenoma with HGD
32*	Inflammatory alterations	Neuroendocrine tumour	Neuroendocrine tumor [pT2, pN0(0/7), G1, V0, L0, Pn0, R0]
33*	Adenoma with LGD	Adenoma, non-invasive	Adenoma with LGD
34*	Adenoma with LGD	Adenoma with LGD	Adenoma with LGD
35*	Adenoma with HGD	Adenoma with LGD	Adenoma with LGD
36*	Duodenum invagination	-	Duodenum invagination
37*	Adenoma with LGD	Adenoma with LGD	Adenoma with LGD

* eVAC-CIP-group

** No preoperative histology: diagnosis obtained at follow-up imaging

*Abbreviations* HGD, high-grade dysplasia; LGD, low-grade dysplasia

## Discussion

To the authors’ knowledge, this is the first series in the literature to report clinical outcomes of prophylactic eVAC combined with post-operative CPI for prevention of bilio-pancreatic leak after tAMP.

Thanks to technological evolution, endoscopic approaches have begun to gain popularity alongside surgical treatment for fistulas and leaks. Therapeutic eVAC has been used with high success rates in decreasing both morbidity and mortality, treating a variety of leaks throughout the GI tract [9, 10]. An emerging concept is to use this technology in the prophylactic setting, thus reducing the anastomotic leak rates and postoperative morbidity. Preemptive eVAC has already been applied in a variety of surgical procedures; although limited to small patient samples, these studies demonstrated the safety of this approach, which may have the potential to improve surgical outcomes in patients receiving visceral anastomoses at high risk of dehiscence [5]. 

Thirty-seven patients underwent surgical tAMP over a ten-year period at our department. Since December 2019 we implemented as routine part of the perioperative approach the prophylactic use of eVAC to prevent anastomotic leak at the reimplantation site of the common bilio-pancreatic ostium. Beside of concept of vacuum application to prevent an anastomosis leakage, we used for the same patients the CPI concept to protect the anastomosis from further damage in case of postoperative pancreatic and biliary fistulas continuously removing pancreatic and biliary enzymes. We have previously reported a significant reduction of clinically relevant postoperative pancreatic fistula (CR-POPF) by applying CPI after major pancreatic resections [8].

In our cohort we experienced a 67% reduction in anastomotic leak in tAMP after introduction of prophylactic eVAC combined with CPI. Moreover, the only leak occurring in the interventional group was managed conservatively, while half of the cases occurring in the historical cohort required surgical revision.

Despite the low sample size of our study prevented us to demonstrate a strong statistical reduction of anastomotic leak by applying prophylactic eVAC and CPI, we indirectly demonstrated a strong trend of reduction of severe post-operative complications by using prophylactic eVAC and CPI. Namely the reduction of acute pancreatitis and the reduction of re-operations or re-interventions due to anastomosis leak, haemorrhage, or abscess formation, which all account for major forms of postoperative morbidity after tAMP [6]. 

The incidence of severe post-operative complications observed in our cohort, also in regard of our historical group, were comparable to most other literature reports [6], despite slightly higher with respect to a recent case series [11]. Our overall mortality rate was zero, which is even lower than in previous reports for this demanding surgery [12]. 

In all our 16 patients who systematically received prophylactic vacuum therapy, we did not encounter any single eVAC-related complication. No dislocation, decubitus or system failure were registered. The presence of prophylactic eVAC was generally well tolerated, with moderate or no discomfort at all for the patients. A potential downside of prophylactic eVAC is the additional time needed for the endoscopic set up and correct positioning of the sponge during the surgical procedure, which required about 30 min more than in the historical cohort. Additionally, prophylactic eVAC necessitates a post-operative drinking restriction of 300 milliliters per day for at least 5 days, requiring parenteral nutrition support for the patient. This is not regularly of need without eVAC.

Collaterally, our cohort confirmed the low accuracy of endoscopic biopsies in ruling-out intra-adenomatous malignancies due to their high false-negative rates [3]. In our population there was disagreement in more than one third of the surgical specimens (35.1%), which were found mostly to contain foci of high-grade dysplasia or even invasive adenocarcinomas within a benign ampullary adenoma diagnosed preoperatively by means of endoscopic biopsies. This finding strengthens the indication for surgical excision in case of ampullary lesion not amenable of endoscopic, complete resection.

Our retrospective, case-control study ranks among the four largest published tAMP series. To our knowledge, it is only slightly surpassed by a center series from Germany [13], Korea [11], and the United States [5]. However, all existing series are small due to the relative rarity of this pathology, together with the limited indications for this approach and the low number of specialized centers performing this procedure worldwide. We acknowledge that the small population of our study cannot be expected to represent a cornerstone in the evidence-generating literature. It is obvious that stronger conclusions and recommendations regarding the real effectiveness of prophylactic eVAC in combination with CPI in reducing bilio-pancreatic leak after tAMP may be drawn only by increasing the population sample. Collecting a consistent number of subjects will be reasonably possible in the context of multicenter trials. We believe that eVAC and CPI should not be tested separately in order to maximize patient safety after this rare and delicate procedure.

## Conclusion

This study is the first to provide technical insights into the feasibility of a prophylactic approach with eVAC along with CPI to reduce anastomotic leak after tAMP. Despite of the limited sample size, we demonstrated a trend of reduction of some major post-operative complications by using eVAC in combination with CPI. Moreover, we did not experience any vacuum-related morbidity, thus proving the safety of this approach for patients undergoing tAMP. We strongly believe that, by increasing our population we will be able to reach enough statistical power to demonstrate the clinical benefits of our perioperative management in patients undergoing this type of surgery.

## Data Availability

Dataset will be provided, in anonymized form, upon reasonable request to the corresponding author.
